# Does MIDAS reduction at 3 months predict the outcome of erenumab treatment? A real-world, open-label trial

**DOI:** 10.1186/s10194-022-01480-2

**Published:** 2022-09-17

**Authors:** Roberto De Icco, Gloria Vaghi, Marta Allena, Natascia Ghiotto, Elena Guaschino, Daniele Martinelli, Lara Ahmad, Michele Corrado, Federico Bighiani, Federica Tanganelli, Sara Bottiroli, Francescantonio Cammarota, Grazia Sances, Cristina Tassorelli

**Affiliations:** 1 Department of Brain and Behavioral Sciences, University of Pavia, Pavia, Italy; 2 Headache Science and Neurorehabilitation Center, IRCCS Mondino Foundation, Pavia, Italy; 3 Faculty of Law, Giustino Fortunato University, Benevento, Italy

**Keywords:** Migraine, Headache, CGRP, Monoclonal antibodies, Pain, Chronic migraine, Disability, Quality of life

## Abstract

**Background:**

In Italy, monoclonal antibodies targeting the CGRP pathway are subsidized for the preventive treatment of high frequency and chronic migraine (CM) in patients with a MIgraine Disability ASsessment (MIDAS) score ≥ 11. Eligibility to treatment continuation requires a ≥ 50% MIDAS score reduction at three months (T3). In this study, we evaluate whether a ≥ 50% MIDAS score reduction at T3 is a reliable predictor of response to one-year erenumab treatment.

**Methods:**

In this prospective, open-label, real-world study, 77 CM patients were treated with erenumab 70–140 mg s.c. every 28 days for one year (T13). We collected the following variables: monthly migraine days (MMDs), monthly headache days (MHDs), days of acute medication intake, MIDAS, HIT-6, anxiety, depression, quality of life and allodynia. Response to erenumab was evaluated as: i) average reduction in MMDs during the 1-year treatment period; and ii) percentage of patients with ≥ 50% reduction in MMDs during the last 4 weeks after the 13^th^ injection (Responders^T13^).

**Results:**

Erenumab induced a sustained reduction in MMDs, MHDs and intake of acute medications across the 12-month treatment period, with 64.9% of patients qualifying as Responders^T13^. At T3, 55.8% of patients reported a ≥ 50% reduction in MIDAS score (MIDAS^Res^) and 55.4% of patients reported a ≥ 50% reduction in MMDs (MMD^Res^). MIDAS^Res^ and MMD^Res^ patients showed a more pronounced reduction in MMDs during the 1-year treatment as compared to NON-MIDAS^Res^ (MIDAS^Res^: T0: 23.5 ± 4.9 vs. T13: 7.7 ± 6.2; NON- MIDAS^Res^: T0: 21.6 ± 5.4 vs. T13: 11.3 ± 8.8, *p* = 0.045) and NON-MMD^Res^ (MMD^Res^: T0: 23.0 ± 4.5 vs. T13: 6.6 ± 4.8; NON-MMD^Res^: T0: 22.3 ± 6.0 vs. T13: 12.7 ± 9.2, *p* < 0.001) groups. The percentage of Responders^T13^ did not differ between MIDAS^Res^ (74.4%) and NON-MIDAS^Res^ (52.9%) patients (*p* = 0.058), while the percentage of Responders^T13^ was higher in the MMD^Res^ group (83.3%) when compared to NON-MMD^Res^ (42.9%) (*p* = 0.001). MMD^Res^ predicted the long-term outcome according to a multivariate analysis (Exp(B) = 7.128; *p* = 0.001), while MIDAS^Res^ did not. Treatment discontinuation based on MIDAS^Res^ would have early excluded 36.0% of Responders^T13^. Discontinuation based on “*either* MIDAS^Res^ *or* MMD^Res^” would have excluded a lower percentage (16%) of Responders^T13^.

**Conclusion:**

MIDAS^Res^ only partly reflects the 12-month outcome of erenumab treatment in CM, as it excludes more than one third of responders. A criterion based on the alternative consideration of ≥ 50% reduction in MIDAS score or MMDs in the first three months of treatment represents a more precise and inclusive option.

**Trial registration:**

The trial was retrospectively registered at www.clinicaltrials.gov (NCT05442008).

**Graphical Abstract:**

CGRP: Calcitonin Gene Related Peptide. MIDAS: MIgraine Disability Assessment. MMDs: monthly migraine days. MIDAS^Res^: Patients with a MIDAS score reduction of at least 50% at T3. MMD^Res^: Patients with a MMDs reduction of at least 50% at T3. Responder^T13^: Patients with a MMDs reduction from baseline of at least 50% in the last 4 weeks of observation period (after 13 erenumab administrations). T0: First erenumab administration. T3, T6, T9, T12: Follow-up visits at three, six, nine, and twelve months after first erenumab administration. T13: Last visit of the protocol.

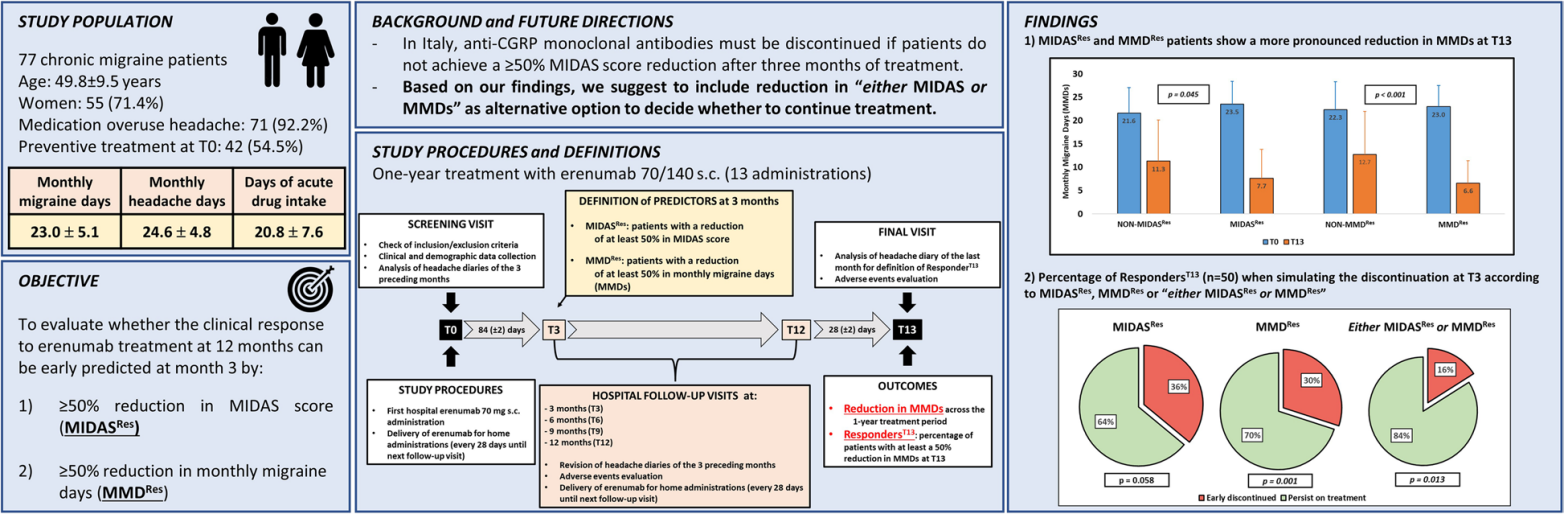

**Supplementary Information:**

The online version contains supplementary material available at 10.1186/s10194-022-01480-2.

## Background

Monoclonal antibodies directed against the Calcitonin Gene Related Peptide pathway (CGRP-mAbs) are a silver lining in the setting of migraine treatment, partly overcoming the issues related to poor effectiveness and tolerability of oral preventive treatments [1].

These drugs act on the CGRP pathway, a key vasoactive neuropeptide in migraine pathophysiology involved in activation and sensitization of afferent trigeminal nociceptors of the trigeminovascular system [2, 3]. Galcanezumab, fremanezumab and eptinezumab target the CGRP ligand, while erenumab, a fully human IgG2, targets its receptor [4]. CGRP-mAbs' efficacy and safety have been largely documented in multiple randomized and open-label studies [5, 6]. Effectiveness and good tolerability has also been supported in the real-world setting [7–10].

In 2019, the Italian Medicines Agency (AIFA) approved subsidization of CGRP-mAbs for the preventive treatment of migraine in patients that satisfy the following criteria: i) at least 8 migraine days per month in the last three months, ii) a MIgraine Disability ASsessment (MIDAS) score ≥ 11, and iii) previous failure due to lack of efficacy or tolerability of at least three preventive drugs, among β-blockers, tricyclic antidepressants, antiepileptics, and onabotulinumtoxin-A (this latter only for chronic migraine (CM)) [11].

After an initial period of three months, the treatment can be continued only in those patients reporting a ≥ 50% reduction in MIDAS score. Thus, in Italy MIDAS score became the driving indicator for treatment initiation and the limiting step for treatment continuation. MIDAS is a self-administered five-item questionnaire that quantifies migraine-related limitation in home and workplace performances. MIDAS score ranges from a minimum of 0 (absence of disability) to a maximum of 270 (indicating an extremely severe disability); a score ≥ 11 identifies patients with a moderate migraine-related disability, while a score ≥ 21 suggests a severe disability [12]. The three-month checkpoint adopted by AIFA is in line with the output of the pivotal RCTs and the subsequent observational studies dealing with CGRP-mAbs in migraine prevention demonstrating the onset of efficacy already in the first weeks after starting treatment [13]. It is also in agreement with the initial EHF guidelines for the use of CGRP-mAbs in migraine prevention [14]. It must however be noted that MIDAS score reduction has not been tested as a predictor of long-term outcome of CGRP-mAbs treatment, although MIDAS was considered as a secondary outcome in several RCTs [5, 15–17]. A major concern for using a single numerical parameter as a mandatory criterion for the initiation and continuation of a preventive treatment is the limited ability to capture the multifaced disability that characterizes the migraine spectrum. On the other side, the reduction in monthly migraine days (MMDs), which represented a primary outcome for clinical trials, may not adequately reflect patients’ preferences and perspectives [18, 19].

Furthermore, real life evidence recently brought to light cases of delayed CGRP-mAbs benefit, worth of a subsequent evaluation after six months of treatment, which questions the validity of a check-point at three months [20].

Due to the high cost of long-term course with CGRP-mAbs, the availability of reliable predictors of outcome is crucial for a careful selection of candidates to start and maintain treatment [21, 22]. The primary aim of this study is to evaluate whether the ≥ 50% reduction in MIDAS score at three months of treatment is predictive of response at 12 months or there are other indicators that might be more suitable for the purpose.

## Methods

### Subjects

We consecutively screened 82 migraine patients (mean age 49.5 ± 9.8 years, 59 females) among those attending the outpatient clinic of the Headache Science & Neurorehabilitation Center of the IRCCS Mondino Foundation (Pavia, Italy) in the period December 2018-January 2020.

The inclusion criteria adopted for selecting patients to be included in the analysis were in agreement with AIFA regulations for CGRP-mAbs prescription and consisted in: age between 18 and 65 years; diagnosis of CM, according to ICHD-3 criteria, for at least 12 months prior to enrollment [23]; prospective completion of a headache diary in the 3 months preceding the enrollment; compliance to complete a daily headache diary for 1 year; previous failure of at least 3 classes of preventive treatments among beta-blockers, antiepileptic drugs, antidepressants, or onabotulinumtoxin-A; a MIDAS score ≥ 11.

Exclusion criteria were: severe cardiologic comorbidities; pregnancy and breastfeeding and previous adverse reaction to latex. A previous therapeutic failure was defined as: i) the lack of efficacy after a 6-week treatment course with an adequate dose, or ii) drug interruption due to poor tolerability or adverse events.

**Fig. 1:**
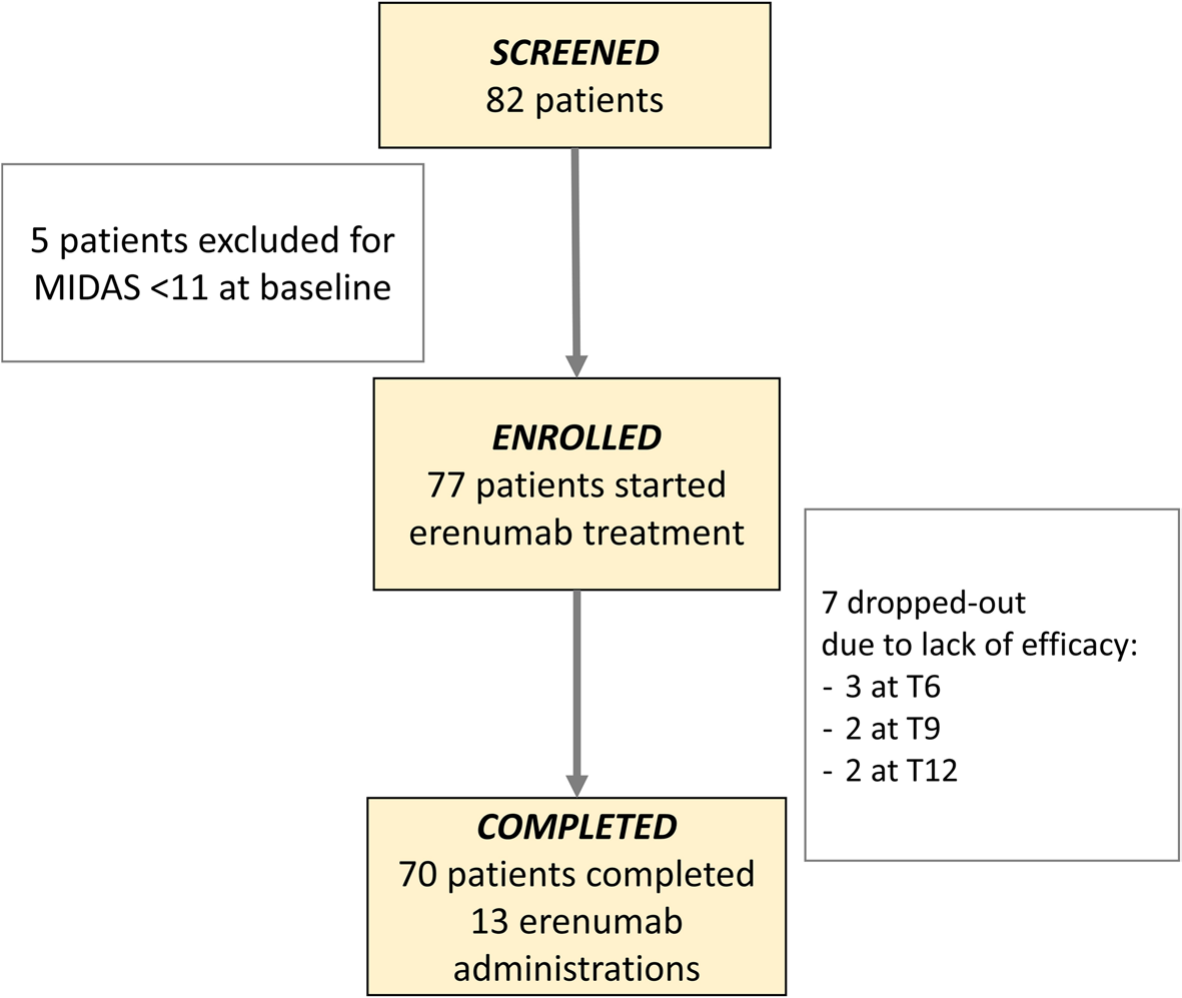
Flowchart of enrolled patients. MIDAS: MIgraine Disability ASsessment. T6, T9, T12: follow-up visits at six, nine, and twelve months after first erenumab administration

Five of the 82 screened patients did not meet inclusion criteria as they presented a baseline MIDAS < 11 and were therefore excluded. The final study population was thus formed by 77 migraine patients (see flowchart in Fig. 1).

### Study procedures

In this prospective, open-label, real-world study, the patients underwent a 1-year treatment with a s.c. erenumab administration every 28 days (13 administrations in total) regardless of MIDAS reduction at three and six months. This was possible due to the temporary dispensation regulations agreed by our Institute with the drug manufacturer, while waiting for AIFA regulations. We used erenumab as it was the first CGRP-mAb that became available in Italy according to this dispensation modality.

At T0, we checked the inclusion/exclusion criteria and the headache diaries of the three preceding months, and we performed a full neurologic and general examination and a thorough anamnestic evaluation.

Patients who agreed to participate in the study signed a written informed consent and completed the baseline procedures, including clinical and demographic data recording and completion of a set of questionnaires to assess migraine related disability/impact (MIDAS and HIT-6), psychological comorbidities (HADS-A and HADS-D), quality of life (MSQ), self-perception of general health (0 to 100 visual analogue scale), and allodynia (ASC-12).

The first erenumab administration (70 mg) was delivered in the hospital setting at T0, where the patients stayed  for a 2-h observation to monitor possible acute adverse events. The patients were then instructed to self-administer the subsequent erenumab doses at home, one every 28 days (T1 to T12).

**Fig. 2:**
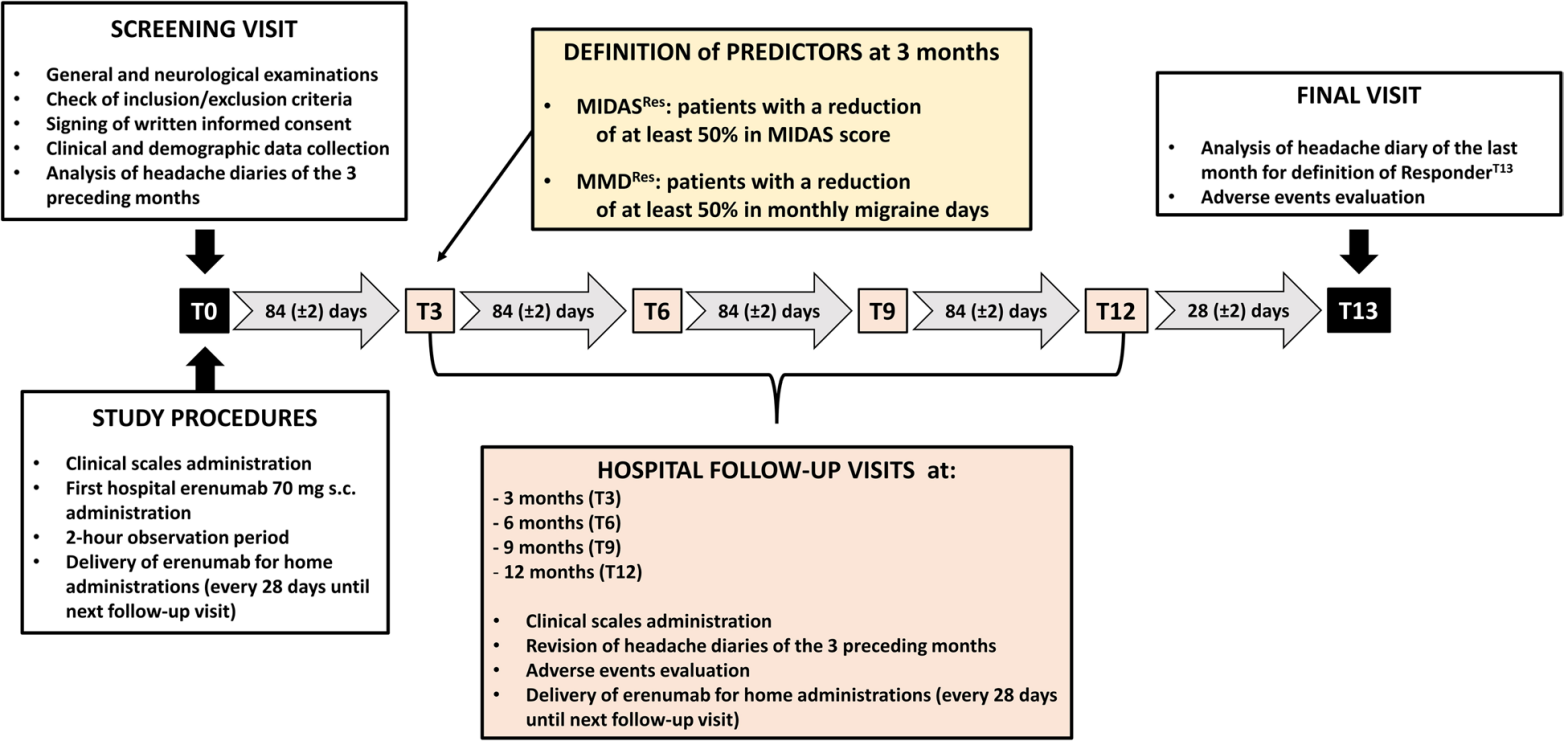
Timeline and study procedures. MIDAS: MIgraine Disability Assessment. MIDAS^Res^: Patients with a MIDAS score reduction of at least 50% at T3. MMD^Res^: Patients with a MMDs reduction of at least 50% at T3. Responders^T13^: Patients with a MMDs reduction from baseline of at least 50% in the last 4 weeks of observation period (after 13 erenumab administrations). T3, T6, T9, T12: follow-up visits at three, six, nine, and twelve months after first erenumab administration

The patients returned to the Center every 12 weeks for the follow-up visits (T3 – T6 – T9 – T12 – Fig. 2), and at T13 for the last visit of the protocol. Monthly headache days (MHDs), monthly migraine days (MMDs), and days and doses of acute drug intake were prospectively recorded in a paper headache diary. At each follow-up visit, the patients completed the same study procedures and clinical scales described for T0. At T3, erenumab dosage was increased to 140 mg in 72 patients based on the clinical response observed in the first three months. More specifically, erenumab dosage was increased to 140 mg in all patients who did not experience a stable reduction in MMDs of at least 75% from baseline. In all of these patients, the dose was then kept stable until the end of the study protocol.

All patients were allowed to keep their oral preventive medications with a stable dose across all study duration.

The study was approved by the local Ethics Committee (P-20190105434) and was registered retrospectively on www.clinicaltrials.gov (NCT05442008).

### Outcomes

Our primary outcome was to evaluate whether a ≥ 50% reduction in the MIDAS score at T3 (MIDAS^Res^) was predictive of a more pronounced reduction in MMDs during a 1-year erenumab treatment. This was calculated from the diaries as the average reduction from baseline observed in the period month 1—month 12. As co-primary measure, we explored the possibility to use a ≥ 50% reduction in MMDs at T3 (MMD^Res^) as an alternative indicator.

We also considered the following secondary outcome measures:

- association between MIDAS^Res^ or MMD^Res^ and the percentage of patients with a ≥ 50% reduction in MMDs in the last 4 weeks of observation period (T13) when compared to baseline (Responders^T13^);
- association between baseline clinical/demographic features and long-term efficacy of erenumab treatment.

Finally, we evaluated the 1-year changes in migraine-related disability, anxiety and depression severity, allodynia, and quality of life as exploratory outcome measures.

### Statistical analysis

The sample size was computed with the freeware online platform www.openepi.com. For the co-primary outcomes, we considered as clinically meaningful a difference between MIDAS^Res^ and NON-MIDAS^Res^ of at least 4 MMDs across the overall study period [15, 24]. Thus, we used the following parameters: confidence interval (2-sided): 95%; power: 80%; ratio of sample size: 2/3 of patients are expected to be in the responder groups; mean difference: 4; standard deviation: 5 for both groups. The minimum suggested sample size was 53 (33 for MIDAS^Res^ and 20 for NON-MIDAS^Res^). To compensate for drop-outs during the 1-year study period and for possible variability in the ratio of sample size, we planned to enroll at least 70 patients with a complete follow-up.

Statistical analysis was conducted with the SPSS software, ver. 21 (IBM Corp., USA) and with “R: A language and environment for statistical computing” (R Foundation for Statistical Computing, Vienna, Austria), Version 1.2.5033, for Windows. Categorical data were reported as absolute numbers and percentages, while continuous variables as mean ± standard deviation.

The Kolmogorov–Smirnov test proved a non-normal distribution of a subset of data (for example MMDs, MHDs, and days of acute drug intake), thus non-parametric tests were used.

For continuous variables, differences between groups were analyzed using the Mann–Whitney U test, while for categorical variables, statistical analysis was performed with the chi-square test.

To evaluate the association between the MIDAS^Res^ and MMD^Res^ status and the modification of MMDs across the 1 year follow-up, we used non-parametric tests for repeated measures with two factors [25]: factor TIME (within subjects, 14 levels: T_0_ T_13_), and factor GROUP (between subjects, 2 levels: MIDAS^Res^ vs. NON-MIDAS^Res^, or MMD^Res^ vs. NON-MMD^Res^).

As exploratory outcomes, we analyzed association between MIDAS^Res^ or MMD^Res^ and HIT-6, HADS-A and HADS-D, ASC-12, MSQ, and self-perceived quality of life; in this case the non-parametric tests for repeated measures included five timepoints (T0, T3, T6, T9, T12). HIT-6, HADS-A and HADS-D, MSQ were available for 43 patients.

Finally, we performed a logistic regression with “Responders^T13^ vs. NON-Responders^T13^” as dependent variable, MIDAS^Res^, MMD^Res^, age, sex as well as other clinical and demographic variables of interest according to the univariate analysis. For all the previously described analyses, the levels of significance were corrected with a Bonferroni method to account for multiple comparison, when necessary.

For all the performed tests, the level of significance was set at α = 0.050.

## Results

### Study population

The final study population included 77 migraine patients (71.4% females, 49.8 ± 9.5 years) with headache onset at 14.5 ± 6.9 years, and a history of CM of 13.1 ± 10.3 years. Of these 77 patients, 7 (9.1%) withdrew from the study between T6 and T12 (due to lack of efficacy) and were considered as drop-outs; according to the planned intention-to-treat analysis, these patients were still included as NON-Responders^T13^.

At baseline, patients reported 23.0 ± 5.1 MMDs and 24.6 ± 4.8 MHDs. Seventy-one out of 77 patients (92.2%) had a concomitant diagnosis of medication overuse headache (MOH). Sixty-nine (89.6%) patients previously underwent at least one in-hospital detoxification procedure. The patients had previously failed 5.1 ± 1.5 preventive medications, and 42 (54.5%) were still taking a preventive treatment at baseline.

The most prevalent comorbidities were anxiety (28.6%), depression (5.2%), or both (23.4%), insomnia (42.9%), or controlled hypertension (24.7%). Clinical and demographic features are detailed in Table 1.

**Table 1:** Clinical and demographic features of study population, and comparison between MIDAS^Res^ / NON-MIDAS^Res^ and MMD^Res^ / NON-MMD^Res^ groups

	**Total**	**MIDAS**^**Res**^	**NON-MIDAS**^**Res**^	***p*-value**	**MMD**^**Res**^	**NON-MMD**^**Res**^	***p*-value**
*n*	77	43	34	-	42	35	-
Age, (years, m ± sd)	49.8 ± 9.5	50.3 ± 9.1	49.2 ± 10.0	0.597	50.0 ± 9.1	49.2 ± 10.0	0.967
Females, *n* (%)	55 (71.4)	32 (74.4)	23 (67.6)	0.614	31 (73.8)	24 (68.6)	0.623
Age at headache onset (years, m ± sd)	14.5 ± 6.9	13.2 ± 5.5	16.2 ± 8.1	0.104	15.2 ± 7.3	16.2 ± 8.1	0.300
Years lived with migraine (years, m ± sd)	35.6 ± 10.8	37.1 ± 9.6	33.6 ± 11.9	0.177	35.3 ± 11.0	33.6 ± 11.9	0.701
Years lived with chronic migraine (years, m ± sd)	13.1 ± 10.3	13.7 ± 10.3	12.4 ± 10.3	0.495	12.0 ± 9.3	12.4 ± 10.3	0.424
Migraine with aura, *n* (%)	13 (16.9)	6 (14.0)	7 (20.6)	0.545	8 (19.0)	5 (14.3)	0.762
MOH, n (%)	71 (92.2)	41 (95.3)	30 (88.2)	0.397	39 (92.9)	32 (91.4)	0.654
Previous detoxification for MOH, *n* (%)	69 (89.6)	40 (93.0)	29 (85.3)		38 (90.5)	32 (91.4)	0.652
Preventive treatment							
Patients on preventive treatment at T0, *n* (%)	42 (54.5)	18 (41.9)	24 (70.6)	**0.021**	24 (57.1)	18 (51.4)	0.652
Number of previously failed preventive treatments (m ± sd)	3.8 ± 1.2	4.0 ± 1.3	3.6 ± 1.5	0.199	3.8 ± 1.6	4.1 ± 0.98	0.439
Previous BoNT-A treatment, *n* (%)	47 (61.0)	27 (62.8)	20 (58.8)	0.815	24 (57.1)	23 (65.7)	0.488
Classes of acute drugs							
NSAIDs, *n* (%)	12 (15.6)	8 (18.6)	4 (11.8)	0.809	7 (16.7)	5 (14.3)	0.457
Triptans, *n* (%)	20 (26.0)	9 (20.9)	11 (32.4)		9 (21.4)	11 (31.4)	
Combination, *n* (%)	4 (5.2)	3 (7.0)	1 (2.9)		1 (2.4)	16 (45.7)	
Multiple drug classes, *n* (%)	41 (53.2)	23 (53.5)	18 (52.9)		25 (59.5)	3 (8.57)	
Comorbidities							
Hypertension, *n* (%)	19 (24.7)	9 (20.9)	10 (29.4)	0.434	11 (26.2)	8 (22.9)	0.795
Anxiety, *n* (%)	22 (28.6)	13 (30.2)	9 (26.5)	0.916	12 (28.6)	10 (28.6)	0.999
Depression, *n* (%)	4 (5.2)	3 (7.0)	1 (2.9)		2 (4.8)	2 (5.7)	
Anxiety and depression, *n* (%)	18 (23.4)	9 (20.9)	9 (26.5)		10 (23.8)	8 (22.9)	
Insomnia, *n* (%)	33 (42.9)	18 (41.9)	15 (44.1)	0.842	18 (42.9)	15 (42.9)	1.000
Migraine features at baseline							
Monthly headache days (m ± sd)	24.6 ± 4.8	25.3 ± 4.2	23.7 ± 5.4	0.281	24.5 ± 4.4	23.7 ± 5.4	0.545
Monthly migraine days (m ± sd)	23.0 ± 5.1	23.7 ± 4.8	22.0 ± 5.3	0.172	23.2 ± 4.5	22.0 ± 5.3	0.861
Monthly days of acute drugs intake (m ± sd)	20.8 ± 7.6	22.8 ± 7.1	18.1 ± 7.5	**0.005**	21.5 ± 6.9	18.1 ± 7.5	0.534
Monthly doses of acute drugs intake (m ± sd)	34.7 ± 29.9	39.9 ± 33.5	28.7 ± 23.5	**0.045**	33.4 ± 29.9	36.4 ± 31.6	0.662
Questionnaires at baseline							
MIDAS (m ± sd)	77.5 ± 69.0	80.9 ± 74.1	73.2 ± 62.6	0.275	72.1 ± 71.8	73.2 ± 62.8	0.452
Mean headache intensity (m ± sd)	8.0 ± 6.4	7.3 ± 1.3	9.0 ± 9.4	0.461	7.3 ± 1.2	9.0 ± 9.4	0.349
HIT-6 (m ± sd)	66.8 ± 5.8	67.1 ± 5.3	66.5 ± 6.4	0.651	65.8 ± 5.7	66.5 ± 6.5	0.113
ASC-12 (allodynia) (m ± sd)	6.4 ± 5.1	5.9 ± 5.4	6.9 ± 4.8	0.264	6.2 ± 5.2	6.9 ± 4.8	0.707
MSQ (m ± sd)	34.4 ± 18.1	38.1 ± 15.4	30.0 ± 20.3	0.068	37.4 ± 14.3	29.6 ± 20.3	0.190
General Health (0–100) (m ± sd)	54.4 ± 22.3	54.2 ± 22.8	54.7 ± 21.9	0.869	61.5 ± 16.6	54.7 ± 22.0	**0.002**
HADS-A (m ± sd)	6.51 ± 4.03	6.1 ± 3.7	7.0 ± 4.4	0.437	5.7 ± 3.6	7.0 ± 4.4	0.079
HADS-D (m ± sd)	6.39 ± 4.40	6.0 ± 5.0	6.9 ± 4.3	0.255	5.4 ± 3.7	6.9 ± 4.3	**0.035**

*Legend:* MIDAS^Res^: Patients with a MIDAS score reduction of at least 50% at T3. NON-MIDAS^Res^: Patients with a MIDAS score reduction < 50% at T3. MMD^Res^: Patients with a MMDs reduction of at least 50% at T3. NON-MMD^Res^: Patients with a MMDs reduction < 50% at T3. T3: follow-up visit at three months after first erenumab administration.

*CM* Chronic migraine, *CM* + *MOH* Chronic migraine and medication overuse headache, *BoNT-A* Onabotulinumtoxin-A, *MIDAS* MIgraine Disability Assessment, *HIT-6* Headache Impact Test-6, *ASC-12* Allodynia Symptoms Checlist, *MSQ* Migraine-Specific Quality of Life Questionnaire, *HADS* Hospital Anxiety and Depression Scale. Data are presented as means ± standard deviations (m ± sd) or absolute values (percentages)

### Clinical modification with erenumab administration

**Fig. 3:**
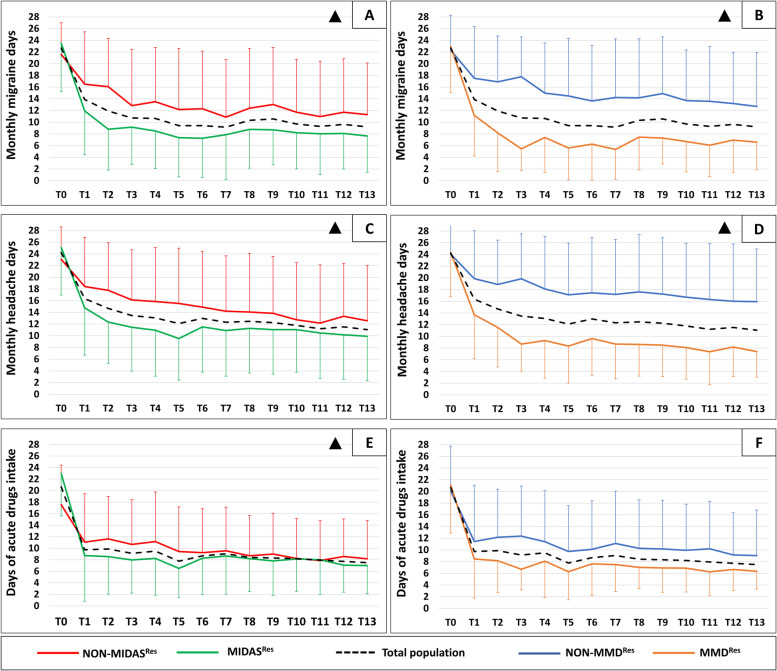
Changes in monthly migraine days, monthly headache days, and days of acute drugs intake in MIDAS^Res^ and MMD^Res^ groups. MIDAS: MIgraine Disability Assessment. MIDAS^Res^ (green lines): Patients with a MIDAS score reduction of at least 50% at T3. NON-MIDAS^Res^ (red lines): Patients with a MIDAS score reduction < 50% at T3. MMD^Res^ (orange lines): Patients with a MMDs reduction of at least 50% at T3. NON-MMD^Res^ (blu lines): Patients with a MMDs reduction < 50% at T3. Black dotted lines represent changes in the overall study population. Δ: TIMExGROUP interaction < 0.050. Statistical analysis was performed with a non-parametric test for repeated measures, with the following factors: TIME: changes in the overall study population across the 1-year treatment period; GROUP: overall difference between study groups; TIMExGROUP interaction: different behavior of study groups across the 1-year treatment period. **A**: difference in monthly migraine days between MIDAS^Res^ and NON-MIDAS^Res^ groups. *TIME* = *p* < *0.001, GROUP* = *p* = *0.034, TIMExGROUP* = *p* = *0.045*. **B**: difference in monthly migraine days between MMD^Res^ and NON-MMD^Res^ groups. *TIME* = *p* < *0.001, GROUP* = *p* < *0.001, TIMExGROUP* = *p* < *0.001.* **C**: difference in monthly headache days between MIDAS^Res^ and NON-MIDAS^Res^ groups. *TIME* = *p* < *0.001,* GROUP = *p* = 0.099, *TIMExGROUP* = *p* = *0.005*. **D**: difference in monthly headache days between MMD^Res^ and NON-MMD^Res^ groups. *TIME* = *p* < *0.001, GROUP* = *p* < *0.001, TIMExGROUP* = *p* < *0.001*. **E**: difference in days of acute drugs intake between MIDAS^Res^ and NON-MIDAS^Res^ groups. TIME = *p* < 0.001, GROUP = *p* = 0.424, TIMExGROUP = *p* = 0.045. **F**: difference in days of acute drugs intake between MMD^Res^ and NON-MMD^Res^ groups. *TIME* = *p* < *0.001*, GROUP = *p* = 0.078, TIMExGROUP = *p* = 0.323

Erenumab induced a sustained reduction in MMDs (T0: 22.7 ± 5.2 days, T1-T13: 10.3 ± 8.2; TIME: F_13,56_ 27.847, *p* < *0.001*), and MHDs (T0: 24.5 ± 4.8, T1-T13: 12.7 ± 8.5; TIME: F_13,56_ 37.622, *p* < *0.001*) (Fig. 3). In line with these results, the days of acute anti-migraine drug intake decreased significantly from T0 to T13 (TIME: F_13,56_ 26.096; *p* < *0.001*). At T13, 50 out of 77 patients (64.9%) qualified as Responders^T13^.

Responders^T13^ were characterized by higher baseline self-perceived general health score (Responders^T13^ 59.2 ± 19.4 vs. NON-Responders^T13^ 45.6 ± 24.9, *p* = *0.022*) and lower baseline HADS-D score (Responders^T13^ 5.6 ± 4.3 vs. NON-Responders^T13^ 7.8 ± 4.3, *p* = *0.031*). No further associations were found with other comorbidities or clinical/demographic features and long-term outcome. Baseline features of Responders^T13^ and NON-Responders^T13^ are detailed in Table 2.

**Table 2:** Clinical and demographic features of Responders^T13^ and NON-Responders^T13^

	**Total**	**Responders**^**T13**^	**NON-Responders**^**T13**^	***p*-value**
*n*	77	50	27	-
Age, (years, m ± sd)	49.8 ± 9.5	49.4 ± 9.4	50.6 ± 9.7	0.967
Females, *n* (%)	55 (71.4)	33 (66.0)	22 (81.5)	0.191
Age at headache onset (years, m ± sd)	14.5 ± 6.9	15.0 ± 7.3	13.6 ± 6.2	0.300
Years lived with migraine (years, m ± sd)	35.6 ± 10.8	34.8 ± 10.5	36.9 ± 11.2	0.701
Years lived with chronic migraine (years, m ± sd)	13.1 ± 10.3	12.6 ± 10.0	14.2 ± 12.6	0.424
Migraine with aura, *n* (%)	13 (16.9)	10 (20.0)	3 (11.1)	0.525
MOH, n (%)	71 (92.2)	40 (95.2)	31 (88.6)	0.402
Previous detoxification for MOH, *n* (%)	69 (89.6)	45 (90.0)	24 (88.9)	0.879
Preventive treatment				
Patients on preventive treatment at T0, *n* (%)	42 (54.5)	28 (56.0)	14 (51.9)	0.812
Number of previously failed preventive treatments (m ± sd)	3.8 ± 1.2	3.7 ± 1.3	4.0 ± 1.0	0.439
Previous BoNT-A treatment, *n* (%)	47 (61.0)	27 (54.0)	20 (74.1)	0.094
Classes of acute drugs				
NSAIDs, *n* (%)	12 (15.6)	7 (16.7)	5 (14.3)	0.408
Triptans, *n* (%)	20 (26.0)	9 (21.4)	11 (31.4)	
Combination, *n* (%)	4 (5.2)	1 (2.4)	3 (8.6)	
Multiple drug classes, *n* (%)	41 (53.2)	25 (59.5)	16 (45.7)	
Comorbidities				
Hypertension, *n* (%)	19 (24.7)	11 (26.2)	8 (22.9)	0.795
Anxiety, *n* (%)	22 (28.6)	12 (28.6)	10 (28.6)	0.999
Depression, *n* (%)	4 (5.2)	2 (4.8)	2 (5.7)	
Anxiety and depression, *n* (%)	18 (23.4)	10 (12.9)	8 (10.3)	
vInsomnia, *n* (%)	33 (42.9)	18 (42.9)	15 (42.9)	1.000
Migraine features at baseline				
Monthly headache days (m ± sd)	24.6 ± 4.8	24.3 ± 4.9	25.2 ± 4.6	0.545
Monthly migraine days (m ± sd)	23.0 ± 5.1	22.9 ± 4.9	23.1 ± 5.4	0.861
Monthly days of acute drugs intake (m ± sd)	20.8 ± 7.6	21.0 ± 7.3	20.3 ± 8.2	0.534
Monthly doses of acute drugs intake (m ± sd)	34.7 ± 29.9	33.8 ± 28.6	36.3 ± 32.6	0.898
Questionnaires at baseline				
MIDAS (m ± sd)	77.5 ± 69.0	74.0 ± 70.7	84.0 ± 66.5	0.452
Mean headache intensity (m ± sd)	8.0 ± 6.4	8.3 ± 7.9	7.5 ± 1.1	0.349
HIT-6 (m ± sd)	66.8 ± 5.8	66.0 ± 5.7	68.3 ± 5.6	0.113
ASC-12 (allodynia) (m ± sd)	6.4 ± 5.1	6.1 ± 5.5	6.8 ± 4.4	0.707
MSQ (m ± sd)	34.4 ± 18.1	36.8 ± 16.0	29.7 ± 21.0	0.190
General Health (0–100) (m ± sd)	54.4 ± 22.3	59.2 ± 19.4	45.6 ± 2 4.9	0.022
HADS-A (m ± sd)	6.51 ± 4.03	6.0 ± 3.9	7.4 ± 4.1	0.079
HADS-D (m ± sd)	6.39 ± 4.40	5.6 ± 4.3	7.8 ± 4.3	0.031

*Legend:* Responders^T13^: Patients with a MMDs reduction from baseline of at least 50% in the last 4 weeks of observation period (after 13 erenumab administrations). NON-Responders^T13^: Patients with a MMDs reduction from baseline < 50% in the last 4 weeks of observation period (after 13 erenumab administrations).

*CM* Chronic migraine, *CM* + *MOH* Chronic migraine and medication overuse headache, *BoNT-A* Onabotulinumtoxin-A, *MIDAS* MIgraine Disability Assessment, *HIT-6* Headache Impact Test-6, *ASC-12* Allodynia Symptoms Checlist, *MSQ* Migraine-Specific Quality of Life Questionnaire, *HADS* Hospital Anxiety and Depression Scale. Data are presented as means ± standard deviations (m ± sd) or absolute values (percentages).

Fourteen (33%) out of 42 patients taking oral preventive therapies at T0 reduced or discontinued these medications during the 1-year erenumab treatment. Fifty out of 77 patients (64.9%) reported at least one adverse event during the study period. The most prevalent adverse events were constipation (46.8%), injection site reactions (10.4%), and fatigue (23.4%). No patient discontinued the treatment due to adverse events (Supplementary Table 1).

### Percentage of MIDAS^Res^ and MMD^Res^ and association with clinical and demographic features

Forty-three out of 77 patients (55.8%) were MIDAS^Res^. MIDAS^Res^ patients were characterized by higher baseline doses of monthly acute drugs (MIDAS^Res^ 39.9 ± 33.5 vs. NON-MIDAS^Res^ 28.7 ± 23.5, *p* = *0.045*) and days of intake (MIDAS^Res^ 22.8 ± 7.1 vs. NON-MIDAS^Res^ 18.1 ± 7.5, *p* = *0.005*) of monthly acute drugs. They were also less likely to take a preventive treatment at baseline (41.9% of MIDAS^Res^ vs. 70.6% of NON-MIDAS^Res^, *p* = *0.021*) (Table 1).

Forty-two out of 77 patients (54.5%) qualified as MMD^Res^. MMD^Res^ patients were characterized by a lower baseline HADS-D score (MMD^Res^ 5.4 ± 3.7 vs. NON-MMD^Res^ 7.5 ± 4.9, *p* = *0.035*), and higher baseline self-perceived general health score (MMD^Res^ 61.5 ± 16.6 vs. non-MMD^Res^ 45.9 ± 25.3, *p* = *0.002*) (Table 1).

Twenty-eight patients (36.4%) qualified as MIDAS^Res^ *and* MMD^Res^, 15 patients (19.5%) were only MIDAS^Res^, 14 patients (18.2%) were only MMD^Res^, and 57 patients (74.0%) were “*either* MIDAS^Res^ *or* MMD^Res^”.

### Predictors of long-term clinical outcome

MIDAS^Res^ patients showed a more pronounced reduction in MMDs during the 1-year treatment as compared to NON- MIDAS^Res^ (GROUP: F_1,68_ 4.503, *p* = *0.034*; and TIMExGROUP: F_13,56_ 1.935, *p* = *0.045*) (Fig. 3). MIDAS^Res^ patients also presented a greater reduction in MHDs and days of acute drug intake during the 1-year treatment (MHDs: GROUP: F_1,68_ 2.705, *p* = *0.099*; and TIMExGROUP: F_13,56_ 2.857, *p* = *0.005*; days of acute drug intake: GROUP: F_1,68_ 0.640, *p* = *0.424*; and TIMExGROUP: F_13,56_ 1.986, *p* = *0.045*) (Supplementary Table 2).

**Fig. 4:**
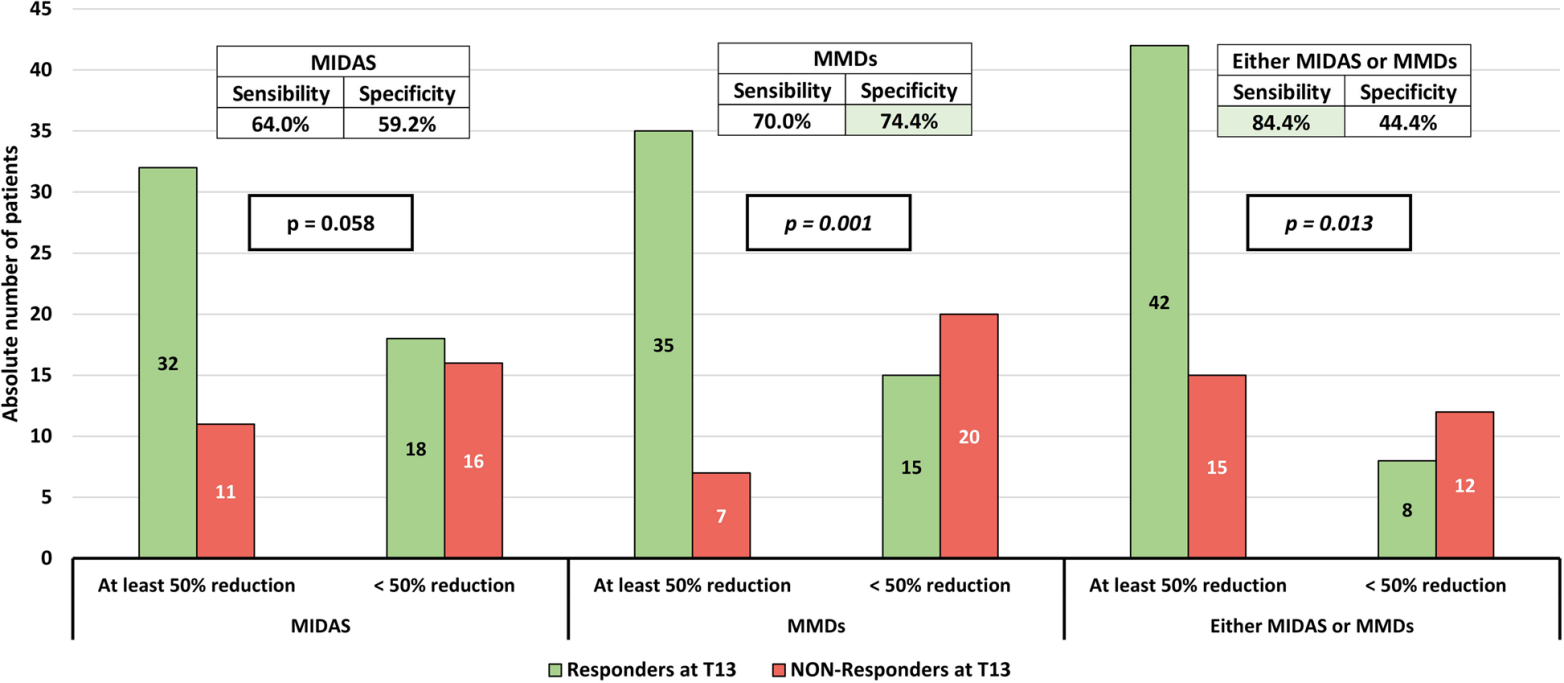
Distribution of Responders^T13^ according to MIDAS and MMDs reductions after 3 months of erenumab administration. MIDAS: MIgraine Disability Assessment. MMDs: monthly migraine days. Responders^T13^: patients with a MMDs reduction from baseline of at least 50% in the last 4 weeks of observation period (after 13 erenumab administrations). The p-value in the box was calculated with a χ^2^ test. The tables in the top of the figure show the sensibility and specificity of being Responders^T13^ according to reduction of at least 50% in MIDAS, MMDs, or "*either* MIDAS *or* MMDs". The green shadows in the tables highlight the parameters with the best accuracy in sensibility or specificity

The percentage of Responders^T13^ did not differ between MIDAS^Res^ (74.4%, 32/43) and NON-MIDAS^Res^ (52.9%, 18/34) patients (*p* = *0.058*) (Fig. 4).

MMD^Res^ patients showed a more pronounced reduction in MMDs during the 1-year treatment as compared to NON-MMD^Res^ (GROUP: F_1,68_ 26.611, *p* < *0.001*; and TIMExGROUP: F_13,56_ 5.476, *p* < *0.001*) (Fig. 3). MMD^Res^ patients also presented a greater reduction in MHDs, but not of days of acute drug intake, during the 1-year treatment (MHDs: GROUP: F_1,68_ 23.881, *p* < *0.001*; and TIMExGROUP: F_13,56_ 6.166, *p* < *0.001*; days of acute drug intake: GROUP: F_1,68_ 3.112, *p* = 0.078; and TIMExGROUP: F_13,56_ 1.159, *p* = 0.323) (Supplementary Table 2).

In addition, a higher percentage of MMD^Res^ (83.3%, 35/42) qualified as Responders^T13^ when compared to NON-MMD^Res^ (42.9%, 15/35) (*p* = *0.001*) (Fig. 4).

The percentage of Responders^T13^ who did not qualify as MIDAS^Res^ or MMD^Res^ was 36.0% and 30.0%, respectively. If we analyze those patients who were “*either* MIDAS^Res^ *or* MMD^Res^” the percentage of early excluded Responders^T13^ markedly decreased to 16% (8/50, *p* = *0.013*) (Fig. 4).

The sensibility and specificity in predicting 1-year erenumab response were: 64% and 59.2% when considering MIDAS^Res^, 70% and 74% when considering MMD^Res^, and 84% and 44.4% when considering “*either* MIDAS^Res^ *or* MMD^Res^” (Fig. 4).

### Multivariate analysis

We conducted a logistic regression analysis using the 1-year clinical outcome (Responders^T13^ vs. non-Responders^T13^) as the dependent variable, and MIDAS^Res^ and MMD^Res^ as covariates, with age and sex included as correction factors. According to the results of the univariate analysis, a stepwise analysis was performed to test the role of baseline general health score, HADS-D score, days of acute drug intake, and use of preventive treatment as possible confounding factors (Table 3).

**Table 3:** Multivariate regression analysis of independent determinants for being Responders^T13^

	aOR = Exp(B)	SE	Wald χ^2^	95% confidence interval	*p* value
Age (years)	0.97	0.03	0.84	0.92–1.0	0.360
Sex	3.6	0.68	3.6	0.96–13.6	0.058
MIDAS^Res^	2.1	0.56	1.8	0.70–6.3	0.185
MMD^Res^	7.1	0.58	11.3	2.3–22.4	**0.001**

*Legend:* aOR: adjusted Odds Ratio; MIDAS^Res^: Patients with a MIDAS reduction of at least 50% at T3. MMD^Res^: Patients with MMDs reduction of at least 50% at T3. +T3: follow-up visit at three months after first erenumab administration. Variables tested but not included in the final model according to a stepwise analysis: baseline general health score, baseline HADS-D score, days of acute drug intake at baseline, and use of preventive treatment at baseline

Using this model, MMD^Res^ was the only predictor of long-term outcome. Specifically, MMD^Res^ patients had 7-time higher odds of being Responders^T13^ (Exp (B) = 7.128; *p* = *0.001*) for the same age, sex, and T3 MIDAS score reduction. The Hosmer–Lemeshow test showed a proper good-of-fit (*p* = *0.687*). This model explained up to 32% (Nagelkerke R^2^) of Response^T13^ variance, classifying our patients correctly in 76.6% of cases.

### Exploratory outcomes

Erenumab induced a sustained improvement in migraine related disability (MIDAS: TIME: F_4,65_ 13.408, *p* < *0.001;* HIT-6: TIME: F_4,65_ 36.665, *p* < *0.001*), severity of anxiety (HADS-A: TIME: F_4,38_ 6.078, *p* = *0.001*) and depression (HADS-D: TIME: F_4,38_ 3.603, *p* < *0.014*), allodynia (ASC-12: TIME: F_4,65_ 6.400, *p* < *0.001*), and quality of life (MSQ: TIME: F_4,38_ 2.996, *p* < *0.030*; self-perceived quality of life: TIME: F_4,38_ 8.581, *p* < *0.001*) (Supplementary Table 3).

When compared to NON-MIDAS^Res^ group, MIDAS^Res^ patients showed a more pronounced improvement in migraine related disability (HIT-6: GROUP: F_1,68_ 2.628, *p* = 0.105; TIMExGROUP: F_4,65_ 4.824, *p* = *0.002*) and quality of life (MSQ: GROUP: F_1,41_ 10.346, *p* = *0.003*; TIMExGROUP: F_4,38_ 5.340, *p* < *0.001*).

When compared to NON-MMD^Res^ group, MMD^Res^ patients showed a greater improvement in quality of life (MSQ: GROUP: F_1,41_ 6.665, *p* = *0.014*; TIMExGROUP: F_4,38_ 3.073, *p* = *0.027*).

## Discussion

In this real-world, open-label, clinical trial, 77 patients with resistant CM were treated with erenumab every 28 days for 1 year. The main objective was to evaluate the performance of MIDAS and MMDs ≥ 50% reduction after 3 months of treatment in predicting the long-term (1-year) clinical effectiveness.

Our findings can be summarized as follows: i) after 3 months of treatment, a similar percentage (about 55%) of patients achieved a ≥ 50% reduction either in MIDAS score (MIDAS^Res^) or MMDs (MMD^Res^); ii) 36% of patients qualified as MIDAS^Res^ *and* MMD^Res^; iii) both MIDAS^Res^ and MMD^Res^ patients showed a greater reduction in MMDs during the 1-year erenumab treatment; and iv) only MMD^Res^ group was associated to the 50% responder outcome at month 13 and this clinical parameter was the only predictor that survived a logistic regression analysis.

In addition, we further confirmed the well-established clinical effectiveness of erenumab. Indeed, we found a sustained reduction in MMDs and days and doses of acute anti-migraine drugs over the 12-month treatment period, together with an improvement in several patients’ reported outcomes. The overall percentage of Responders^T13^ was 65%.

The rate of Responders^T13^ between MIDAS^Res^ and NON-MIDAS^Res^ patients was not statistically significant, although we detected a trend towards a difference. At present, we cannot exclude that this observation may be related to an insufficient statistical power for this secondary outcome and larger cohort studies are needed to confirm this result.

The cost of CGRP-mAbs has required payors to put in place specific regulations [26]. We are however aware that migraine is a social disease that affects people during the most productive years of their life, leading to a huge economic burden due to health care resources exploitation, presenteeism and absenteeism [27, 28]. Thus, a clinical improvement directly translates in a reduction in direct and indirect costs. This is corroborated by a recent study that demonstrated a reduction in healthcare costs in patients with a long-term oral preventive treatment adherence [29]. We feel it is important to provide evidence-based data on novel drugs to guide local as well as global regulations in the decision-making process.

Based on the robust body of evidence, the European Headache Federation (EHF) has recently updated its guidelines for the use of CGRP-mAbs in the prevention of migraine, based on the GRADE system. These evidence-based recommendations confirmed the moderate-to-high quality evidence on the efficacy and safety of CGRP-mAbs in migraine prevention and proposed to include these drugs among first line preventive therapies [20].

A pivotal finding in our cohort, is that up to 36% of patients qualifying as responders at month 13 would have been discontinued early with the application of the present ≥ 50% MIDAS reduction criterion. The same would also be the case if we considered the MMD^Res^ parameter. By contrast, the combination of these two parameters provides an improved accuracy. Indeed, if we applied the “*either* MIDAS^Res^ *or* MMD^Res^” criterion, only 16% of Responders^T13^ would have been discontinued after only 3 months of erenumab treatment.

A recent study by Iannone et al. suggested that the ≥ 50% MIDAS reduction may represent an advantageous outcome measure, as it allows the highest proportion of difficult-to-treat migraine patients to persist on CGRP-mAbs treatment [15]. In their study, the percentage of patients achieving a ≥ 50% MIDAS reduction ranged between 63.5% and 96.1%, which is higher than our experience (55%). The study by Iannone et al. differs from our trial in several aspects: i) first of all, their patients had different follow-up periods, and discontinued treatment at month 3 according to the present AIFA criteria; ii) the study included patients treated with the three available CGRP-mAbs; iii) out of 203 patients enrolled, only 52 completed a 12-month follow-up, and a lower number (*n* = 33) completed a 12-month treatment period, thus analysis of predictors of response could not be projected beyond the 6-month horizon; and iv) the effectiveness of MIDAS score reduction as predictor of clinical outcome was not the primary outcome of the study. In our cohort, we planned to treat all patients for 12 consecutive months, independently of MIDAS score reduction. Only 7 patients were discontinued (9.1%) because they withdrew consent to participate due to lack of efficacy; these patients withdrew after T3, so we were able to record MIDAS modification at this time-point, and all of them were included in the analysis as NON-Responders^T13^.

The overall clinical improvement observed in the present study is in line, or even better, when compared with previous double blind clinical trials, open-label extension trials and real-world evidence. Tepper et al. demonstrated a reduction of 6.6 MMDs after 3 months of erenumab treatment, with a 50% responder rate of 40% [30]. In the subsequent 52-week open-label, MMDs decreased by 9.3 from baseline, and the percentage of patients qualifying as 50% responders was 59% [31]. In the real-world setting, the percentage of 50% responders for CM ranged between 34 and 75%, while the reduction in MMDs varied from 9.3 to 12.8, a quite ample heterogeneity that can be explained by differences in study design, duration of follow-up, and inclusion/exclusion criteria [9, 10, 32, 33].

Identification of clinical, biological or instrumental predictors of outcome represents a top priority in medicine.

Precision and tailored medicine will improve patients’ management as well as resources allocation, but solid predictors of outcome for CGRP-mAbs treatment are lacking so far, although several parameters have been proposed. Some studies suggest that a lower baseline MIDAS score may predict the short- and long-term erenumab response [34–36]. Lower baseline MMDs, short history of chronification, comorbidity with medication overuse headache (MOH), good response to triptans, unilateral pain, cutaneous allodynia, dopaminergic symptoms and spinal central sensitization were proposed as long-term predictors of CGRP-mAbs response [9, 15, 35–39]. Also higher basal headache frequency was associated to a better erenumab response in CM [9]. At variance, psychological and psychiatric comorbidities as well as previously failed preventive therapies seem to play an important role as negative predictors of outcome [7–10, 34–38]. No concordance was found on gender and erenumab responsiveness in CM, as both an association to male sex and no difference among gender were detected [10, 40].

In our analysis, baseline self-perceived general health and lower HADS-D scores were positively associated with Responders^T13^ status. In a logistic regression analysis, only MMD^Res^ group had 7-time higher odds of being Responders^T13^, even when corrected for age, sex, and MIDAS^Res^ status. It is also true that an extensive evaluation of predictors of outcome was beyond the aims of our study. Thus, further analyses are needed to implement an accurate characterization of Responders^T13^ and NON-Responders^T13^ groups as it represents a key-point in the decision-making process and, by far, little concordance is demonstrated among studies.

AIFA selection of the ≥ 50% reduction in MIDAS score at 3 months may be considered a reasonable approach to the issue of CGRP-mAbs subsidization but it lacks scientific evidence [15]. The inclusion/exclusion criteria of the RCTs where CGRP-mAbs proved effective did not consider baseline MIDAS score [24]. The criteria adopted by AIFA for identifying the migraine patients who qualify for CGRP-mAbs subsidization include multiple previous failure of preventive treatments, in analogy to the EHF guidelines for resistant migraine, but these latter did not take into consideration MIDAS score as a driver of treatment duration [41]. MIDAS score was also not considered in the first and second edition of the EHF expert opinion based guidelines for the use of CGRP-mAbs in clinical practice [14, 20], while it is included in the guidelines of the American Headache Society but among a large number of other indicators, which ensures that the physician can use clinical judgement, as expected from our profession [42]. Two last observations are worth consideration. Firstly, the reduction in MIDAS score has never been tested as a predictor of outcome before CGRP-mAbs approval in Italy, and secondly, MIDAS score reduction is not a mandatory decision-making step in other European countries. The first point is crucial, as it prevents testing the effectiveness of CGRP-mAbs in the cohort of patients who do not meet the MIDAS^Res^ status. The second one is also important because it creates disparity in the access to care across Europe and beyond. We believe that the present findings are amenable to a wider application outside of the Italian boundaries to foster the creation of a European or globally shared approach that overcomes differences in local regulations to provide the best care solutions to migraine patients.

Ideally, the best clinical approach for the physicians is not to be forced by a single parameter in the decision to prolong or interrupt CGRP-mAbs therapy, in line with the conventional oral preventive drugs. An isolated parameter cannot reflect the clinical improvement in its complexity, as it depends on several features, namely: reduction in headache frequency, acute drugs consumption, severity of pain levels and most bothersome symptoms as well as improvement in patients’ reported outcomes such as disability, quality of life, working productivity and so on. Pain is a subjective experience that is difficult to assess thoroughly. It is well known that there are patients who may be satisfied by preventive treatments just by experiencing a reduction in pain and associated symptoms intensity [19]. If a check-point step is required to reduce costs associated to CGRP-mAbs, this should be built so as to guarantee treatment to all patients who can benefit.

We acknowledge that our study has some limitations. First of all, we explored the role of MIDAS and MMD reduction as predictors of outcome. As such, we cannot exclude that other clinical parameters, or their combination, might yield a better sensibility and/or specificity. In addition, we did not explore predictors at later points of treatment, as we wanted to focus on the earliest time-point for CGRP-mAbs discontinuation. Although based on a sample size calculation, the result obtained in a population of 77 patients may require replication studies on larger cohorts for confirmation and wider transferability. Our findings are limited to a CM treated with erenumab, which prevents generalization to high frequency episodic migraine or to other anti-CGRP drugs. Other measures, namely reduction in moderate to severe headache days or 30% MMD reduction, may also be considered as clinical outcomes, but we decided to stick to the most recent guidelines for clinical trials in CM [18].

## Conclusions

The present data questions the suitability of a ≥ 50% MIDAS score reduction at 3 months as a requirement for the continuation of CGRP-mAbs treatment in patients with CM based on the following evidence: i) the indicator is poorly associated with the long-term responder rate, ii) it has low sensibility and specificity, and iii) it was outperformed by MMDs^Res^, which showed better sensibility, with acceptable specificity.

However, when considering that CM is one of the most debilitating diseases and that the patients qualifying for the CGRP-mAbs treatment must have endured multiple previous failures of preventive treatments, we believe that a more inclusive and conservative indicator should be adopted to guide treatment continuation: our findings suggest that the “*either* MIDAS^Res^ *or* MMD^Res^” indicator is a valid alternative, as it showed the highest degree of sensibility in identifying responders at month 12.

## Supplementary Information

**Additional file 1:** **Supplementary Table 1**. Drop-outs and adverse event reporting. **Supplementary Table 2.** Monthly headache days, monthly migraine days and days of acute drug intake according to MIDAS and monthly migraine days (MMDs) response at T3. **Supplementary Table 3. **Patients’ reported outcomes according to MIDAS and monthly migraine days (MMDs) response at T3.

## Data Availability

The dataset generated and/or analysed during the current study is available in the Zenodo repository, with the 10.1186/s10194-022-01480-2. The dataset is available from the corresponding author on reasonable request.

## Abbreviations

CGRP: Calcitonin Gene Related Peptide
CGRP-mAbs: Monoclonal antibodies targeting the CGRP pathway
CM: Chronic Migraine
MIDAS: MIgraine Disability ASsessment
MIDAS^Res^: Patients with a MIDAS score reduction of at least 50% at T3
NON-MIDAS^Res^: Patients with a MIDAS score reduction < 50% at T3
MMDs: Monthly Migraine Days
MMD^Res^: Patients with a MMDs reduction of at least 50% at T3
NON-MMD^Res^: Patients with a MMDs reduction < 50% at T3
MHDs: Monthly Headache Days
MOH: Medication Overuse Headache
Responders^T13^: Patients with a MMDs reduction from baseline of at least 50% in the last 4 weeks of observation period (after 13 erenumab administrations)
NON-Responders^T13^: Patients with a MMDs reduction from baseline < 50% in the last 4 weeks of observation period (after 13 erenumab administrations)
