# Supplementary material for: Does MIDAS reduction at 3 months predict the outcome of erenumab treatment? A real-world, open-label trial
Source: J Headache Pain. 2022 Sep 17;23(1):123. doi: 10.1186/s10194-022-01480-2 (PMC9482180; doi:10.1186/s10194-022-01480-2)
Supplement: Supplementary file 1 — Additional file 1: Supplementary Table 1. Drop-outs and adverse event reporting. Supplementary Table 2. Monthly headache days, monthly migraine days and days of acute drug intake according to MIDAS and monthly migraine days (MMDs) response at T3. Supplementary Table 3. Patients’ reported outcomes according to MIDAS and monthly migraine days (MMDs) response at T3. [file 10194_2022_1480_MOESM1_ESM.docx]

**Supplementary Materials**

**Supplementary Table 1** – Drop-outs and adverse event reporting

|  | Drop-outs for any reason | Patients reporting at least one adverse event | Constipation | Injection site reactions | Fatigue | Others |
| --- | --- | --- | --- | --- | --- | --- |
| T1, n (%) | 0/77 (0.0) | 4 (5.2) | 2 (2.6) | 1 (1.3) | 1 (1.3) | 2 (2.6) |
| T3, n (%) | 0/77 (0.0) | 43 (55.8) | 29 (37.7) | 4 (5.2) | 14 (18.2) | 3 (3.9) |
| T6, n (%) | 3/77 (3.9) | 36 (48.9) | 23 (31.1) | 4 (5.4) | 14 (18.9) | 2 (2.7) |
| T9, n (%) | 2/74 (2.7) | 36 (50.0) | 27 (37.5) | 3 (4.2) | 11 (15.3) | 3 (4.2) |
| T12, n (%) | 2/72 (2.8) | 35 (50.0) | 26 (37.1) | 6 (8.6) | 9 (12.9) | 2 (2.9) |
| TOT | 7/77 (9.1) | 50/77 (64.9) | 46 (59.7) | 7 (9.1) | 18 (23.4) | 5 (6.5) |

*Legend:* T1, T3, T6, T9, T12: follow-up visits at one, three, six, nine, and twelve months after first erenumab administration.

**Supplementary Table 2 -** Monthly headache days, monthly migraine days and days of acute drug intake according to MIDAS and monthly migraine days (MMDs) response at T3.

|  | MIDAS^Res^ | NON-MIDAS^Res^ | MMD^Res^ | NON-MMD^Res^ |
| --- | --- | --- | --- | --- |
| n | 43 | 34 | 42 | 35 |
| Monthly headache days | | | | |
| T0 | 25.1 ± 4.3 | 23.1 ± 5.5 | 24.4 ± 4.5 | 24.1 ± 5.5 |
| T1 | 14.8 ± 8.1 | 18.4 ± 8.4 | 13.7 ± 7.5 | 19.9 ± 8.2 |
| T2 | 12.4 ± 7.1 | 17.8 ± 8.1 | 11.5 ± 6.8 | 18.9 ± 7.6 |
| T3 | 11.5 ± 7.5 | 16.1 ± 8.6 | 8.7 ± 4.7 | 19.8 ± 7.7 |
| T4 | 11.0 ± 7.8 | 15.9 ± 9.2 | 9.3 ± 6.4 | 18.1 ± 9.0 |
| T5 | 9.5 ± 7.1 | 15.5 ± 9.4 | 8.4 ± 6.4 | 17.1 ± 8.9 |
| T6 | 11.5 ± 7.4 | 14.9 ± 9.6 | 9.6 ± 6.3 | 17.4 ± 9.5 |
| T7 | 10.9 ± 7.8 | 14.2 ± 9.5 | 8.7 ± 5.9 | 17.2 ± 9.4 |
| T8 | 11.3 ± 7.6 | 14.1 ± 10.0 | 8.6 ± 5.4 | 17.6 ± 9.8 |
| T9 | 11.1 ± 7.6 | 13.8 ± 9.7 | 8.5 ± 5.4 | 17.2 ± 9.6 |
| T10 | 11.1 ± 7.3 | 12.7 ± 9.8 | 8.1 ± 5.4 | 16.7 ± 9.2 |
| T11 | 10.5 ± 7.7 | 12.2 ± 10.0 | 7.4 ± 5.7 | 16.3 ± 9.6 |
| T12 | 10.2 ± 7.6 | 13.3 ± 9.1 | 8.2 ± 5.0 | 16.0 ± 9.8 |
| T13 | 9.9 ± 7.6 | 12.6 ± 9.5 | 7.4 ± 4.4 | 16.0 ± 10.2 |
| Monthly migraine days | | | | |
| T0 | 23.5 ± 4.9 | 21.6 ± 5.4 | 23.0 ± 4.5 | 22.3 ± 6.0 |
| T1 | 11.9 ± 7.4 | 16.5 ± 8.9 | 11.2 ± 6.9 | 17.5 ± 8.9 |
| T2 | 8.8 ± 7.0 | 16.1 ± 8.3 | 8.2 ± 6.6 | 16.9 ± 7.8 |
| T3 | 9.2 ± 6.4 | 12.8 ± 9.6 | 5.5 ± 3.7 | 17.7 ± 6.8 |
| T4 | 8.5 ± 6.4 | 13.5 ± 9.3 | 7.4 ± 6.0 | 15.0 ± 8.6 |
| T5 | 7.4 ± 6.7 | 12.2 ± 10.5 | 5.6 ± 5.5 | 14.5 ± 9.8 |
| T6 | 7.3 ± 6.7 | 12.3 ± 9.8 | 6.3 ± 6.1 | 13.6 ± 9.5 |
| T7 | 7.9 ± 7.7 | 10.9 ± 9.9 | 5.4 ± 5.1 | 14.2 ± 10.0 |
| T8 | 8.8 ± 6.6 | 12.4 ± 10.2 | 7.5 ± 5.6 | 14.2 ± 10.1 |
| T9 | 8.7 ± 6.0 | 13.0 ± 9.7 | 7.3 ± 4.4 | 14.9 ± 9.7 |
| T10 | 8.2 ± 6.2 | 11.7 ± 9.0 | 6.7 ± 5.2 | 13.7 ± 8.7 |
| T11 | 8.0 ± 7.0 | 11.0 ± 9.5 | 6.1 ± 5.4 | 13.6 ± 9.4 |
| T12 | 8.1 ± 6.1 | 11.7 ± 9.1 | 7.0 ± 5.5 | 13.2 ± 8.8 |
| T13 | 7.7 ± 6.2 | 11.3 ± 8.8 | 6.6 ± 4.8 | 12.7 ± 9.2 |
| Days of anti-migraine drugs intake | | | | |
| T0 | 23.0 ± 6.6 | 17.6 ± 6.9 | 21.1 ± 6.8 | 20.1 ± 7.7 |
| T1 | 8.7 ± 7.9 | 11.1 ± 8.4 | 8.5 ± 6.7 | 11.4 ± 9.6 |
| T2 | 8.6 ± 6.5 | 11.6 ± 7.4 | 8.2 ± 5.4 | 12.2 ± 8.2 |
| T3 | 8.0 ± 5.7 | 10.7 ± 7.8 | 6.7 ± 3.5 | 12.4 ± 8.6 |
| T4 | 8.3 ± 6.4 | 11.2 ± 8.6 | 8.1 ± 6.2 | 11.4 ± 8.7 |
| T5 | 6.5 ± 5.0 | 9.4 ± 7.7 | 6.3 ± 4.8 | 9.7 ± 7.8 |
| T6 | 8.3 ± 6.3 | 9.2 ± 7.6 | 7.6 ± 5.4 | 10.1 ± 8.3 |
| T7 | 8.7 ± 6.6 | 9.6 ± 7.5 | 7.5 ± 4.6 | 11.1 ± 9.0 |
| T8 | 8.2 ± 5.7 | 8.7 ± 7.0 | 7.0 ± 3.6 | 10.3 ± 8.3 |
| T9 | 7.8 ± 6.0 | 9.0 ± 7.1 | 6.9 ± 4.2 | 10.2 ± 8.3 |
| T10 | 8.2 ± 5.6 | 8.2 ± 6.9 | 6.9 ± 4.1 | 9.9 ± 7.9 |
| T11 | 8.0 ± 6.0 | 7.9 ± 6.9 | 6.3 ± 4.1 | 10.2 ± 8.1 |
| T12 | 7.1 ± 4.7 | 8.6 ± 6.5 | 6.7 ± 3.6 | 9.1 ± 7.2 |
| T13 | 7.0 ± 4.9 | 8.2 ± 6.7 | 6.4 ± 3.0 | 9.0 ± 7.8 |

*Legend:* data are reported as means ± standard deviation. MIDAS^Res^: Patients with a MIDAS score reduction of at least 50% at T3. NON-MIDAS^Res^: Patients with a MIDAS score reduction <50% at T3. MMD^Res^: Patients with a MMDs reduction of at least 50% at T3. NON-MMD^Res^: Patients with a MMDs reduction <50% at T3. T3: follow-up visit at three months after first erenumab administration. T0: baseline visit. T1 to T13: monthly follow-up visits.

**Supplementary Table 3 –** Patients’ reported outcomes according to MIDAS and monthly migraine days (MMDs) response at T3.

|  | MIDAS^Res^ | NON-MIDAS^Res^ | MMD^Res^ | NON-MMD^Res^ |
| --- | --- | --- | --- | --- |
| n | 43 | 34 | 42 | 35 |
| MIDAS score | | | | |
| T0 | 25.1 ± 4.3 | 23.1 ± 5.5 | 24.4 ± 4.5 | 24.1 ± 5.5 |
| T3 | 11.5 ± 7.5 | 16.1 ± 8.6 | 8.7 ± 4.7 | 19.8 ± 7.7 |
| T6 | 11.5 ± 7.4 | 14.9 ± 9.6 | 9.6 ± 6.3 | 17.4 ± 9.5 |
| T9 | 11.1 ± 7.6 | 13.8 ± 9.7 | 8.5 ± 5.4 | 17.2 ± 9.6 |
| T12 | 10.2 ± 7.6 | 13.3 ± 9.1 | 8.2 ± 5.0 | 16.0 ± 9.8 |
| HIT-6 | | | | |
| T0 | 67.1 ± 5.5 | 66.5 ± 6.7 | 65.9 ± 5.7 | 68.9 ± 6.3 |
| T3 | 57.5 ± 6.0 | 63.5 ± 7.2 | 58.8 ± 6.5 | 63.4 ± 7.8 |
| T6 | 55.4 ± 6.9 | 56.6 ± 8.9 | 55.9 ± 7.8 | 55.7 ± 10.7 |
| T9 | 55.2 ± 8.8 | 57.2 ± 7.8 | 55.2 ± 7.9 | 58.1 ± 9.1 |
| T12 | 55.4 ± 7.7 | 57.2 ± 6.6 | 54.6 ± 7.4 | 59.9 ± 5.5 |
| ASC-12 | | | | |
| T0 | 6.1 ± 5.5 | 6.68 ± 4.9 | 6.1 ± 5.5 | 6.8 ± 4.5 |
| T3 | 4.1 ± 5.5 | 5.7 ± 5.1 | 4.4 ± 5.6 | 5.67 ± 4.8 |
| T6 | 3.1 ± 4.5 | 4.8 ± 4.5 | 3.4 ± 4.4 | 5.0 ± 4.8 |
| T9 | 3.5 ± 4.4 | 4.9 ± 4.3 | 3.6 ± 4.3 | 5.2 ± 4.6 |
| T12 | 2.7 ± 3.7 | 5.2 ± 4.9 | 3.12 ± 4.3 | 5.4 ± 4.4 |
| HADS-A | | | | |
| T0 | 6.4 ± 3.6 | 8.4 ± 3.9 | 6.2 ± 3.4 | 9.8 ± 3.8 |
| T3 | 4.5 ± 2.9 | 8.6 ± 3.5 | 5.3 ± 3.5 | 8.7 ± 3.5 |
| T6 | 5.3 ± 3.7 | 7.9 ± 4.7 | 5.4 ± 3.5 | 9 ± 5.1 |
| T9 | 4.3 ± 3.5 | 5.7 ± 4.6 | 3.4 ± 2.3 | 8.4 ± 5.1 |
| T12 | 5.1 ± 3.8 | 6.8 ± 4.6 | 4.4 ± 3.1 | 9 ± 4.8 |
| HADS-D | | | | |
| T0 | 5.7 ± 4.4 | 8.2 ± 4.1 | 5.9 ± 4.1 | 8.6 ± 4.6 |
| T3 | 4.1 ± 4.1 | 7.9 ± 5.4 | 5.3 ± 5.1 | 6.9 ± 4.8 |
| T6 | 4.4 ± 4.4 | 6.6 ± 4.9 | 4.1 ± 3.2 | 8.5 ± 6.1 |
| T9 | 4.00 ± 4.9 | 5.9 ± 4.6 | 3.53 ± 3.3 | 7.92 ± 6.3 |
| T12 | 4.5 ± 4.5 | 6.3 ± 4.4 | 4.1 ± 3 | 8.1 ± 5.9 |
| MSQ | | | | |
| T0 | 51.46 ± 10.8 | 60.4 ± 15.3 | 54 ± 11.9 | 58.7 ± 16.8 |
| T3 | 62.3 ± 11.7 | 36.8 ± 19.4 | 52.7 ± 20.4 | 47.2 ± 19.4 |
| T6 | 64.7 ± 13.7 | 50.1 ± 15.1 | 61.9 ± 12.5 | 49.9 ± 20.1 |
| T9 | 63.6 ± 17.5 | 56.9 ± 15.7 | 64.4 ± 14.1 | 52.2 ± 20.1 |
| T12 | 63.4 ± 15.3 | 53.63 ± 17.4 | 64.5 ± 11.7 | 46.6 ± 20.2 |
| General Health (0-100) | | | | |
| T0 | 57.1 ± 17.6 | 51.6 ± 21.5 | 59.2 ± 17.3 | 44.2 ± 20.7 |
| T3 | 67.7 ± 18.1 | 55.5 ± 18.6 | 68 ± 15.7 | 49.2 ± 20.5 |
| T6 | 73.2 ± 18.3 | 60.0 ± 26.9 | 73.3 ± 20.4 | 53.8 ± 24.5 |
| T9 | 73.5 ± 19.4 | 71.0 ± 18.1 | 78.8 ± 11.2 | 57.5 ± 23.9 |
| T12 | 74.3 ± 22.9 | 68.0 ± 18.7 | 79.9 ± 11.1 | 52 ± 26.1 |

*Legend:* data are reported as means ± standard deviation. MIDAS^Res^: Patients with a MIDAS score reduction of at least 50% at T3. NON-MIDAS^Res^: Patients with a MIDAS score reduction <50% at T3. MMD^Res^: Patients with a MMDs reduction of at least 50% at T3. NON-MMD^Res^: Patients with a MMDs reduction <50% at T3. T3, T6, T9, T12: follow-up visits at three, six, nine, and twelve months after first erenumab administration. MIDAS: MIgraine Disability Assessment; HIT-6: Headache Impact Test-6; ASC-12: Allodynia Symptoms Checlist; MSQ: Migraine-Specific Quality of Life Questionnaire; HADS: Hospital Anxiety and Depression Scale.
